# Transcriptome-wide characterization, evolutionary analysis, and expression pattern analysis of the NF-Y transcription factor gene family and salt stress response in *Panax ginseng*

**DOI:** 10.1186/s12870-022-03687-6

**Published:** 2022-07-04

**Authors:** Mingming Liu, Zhaoxi Pan, Jie Yu, Lei Zhu, Mingzhu Zhao, Yanfang Wang, Ping Chen, Chang Liu, Jian Hu, Tao Liu, Kangyu Wang, Yi Wang, Meiping Zhang

**Affiliations:** 1 College of Life Science, Jilin Agricultural University, Changchun, 130118 Jilin China; 2 Jilin Engineering Research Center Ginseng Genetic Resources Development and Utilization, Changchun, 130118 Jilin China; 3 College of Chinese Medicinal Materials, Jilin Agricultural University, Changchun, 130118 Jilin China

**Keywords:** *Panax ginseng*, NF-Y transcription factor, Phylogeny, Expression pattern, Salt stress

## Abstract

Jilin ginseng (*Panax ginseng* C. A. Meyer) has a long history of medicinal use worldwide. The quality of ginseng is governed by a variety of internal and external factors. Nuclear factor Y (NF-Y), an important transcription factor in eukaryotes, plays a crucial role in the plant response to abiotic stresses by binding to a specific promoter, the CCAAT box. However, the NF-Y gene family has not been reported in *Panax ginseng*. In this study, 115 *PgNF-Y* transcripts with 40 gene IDs were identified from the Jilin ginseng transcriptome database. These genes were classified into the PgNF-YA (13), PgNF-YB (14), and PgNF-YC (13) subgroups according to their subunit types, and their nucleotide sequence lengths, structural domain information, and amino acid sequence lengths were analyzed. The phylogenetic analysis showed that the 79 *PgNF-Y* transcripts with complete ORFs were divided into three subfamilies, NF-YA, NF-YB, and NF-YC. *PgNF-Y* was annotated to eight subclasses under three major functions (BP, MF, and CC) by GO annotation, indicating that these transcripts perform different functions in ginseng growth and development. Expression pattern analysis of the roots of 42 farm cultivars, 14 different tissues of 4-year-old ginseng plants, and the roots of 4 different-ages of ginseng plants showed that *PgNF-Y* gene expression differed across lineages and had spatiotemporal specificity. Coexpression network analysis showed that PgNF-Ys acted synergistically with each other in Jilin ginseng. In addition, the analysis of the response of *PgNF-YB09*, *PgNF-YC02*, and *PgNF-YC07-04* genes to salt stress treatment was investigated by fluorescence quantitative PCR. The expression of these genes increased after salt stress treatment, indicating that they may be involved in the regulation of the response to salt stresses in ginseng. These results provide important functional genetic resources for the improvement and gene breeding of ginseng in the future.

**Conclusions:** This study fills a knowledge gap regarding the *NF-Y* gene family in ginseng, provides systematic theoretical support for subsequent research on *PgNF-Y* genes, and provides data resources for resistance to salt stress in ginseng.

**Supplementary Information:**

The online version contains supplementary material available at 10.1186/s12870-022-03687-6.

## Background

Nuclear factor Y (NF-Y) family transcription factors are important regulators of plant development and physiology [1]. Nuclear factor Y transcription factors (also known as heme-associated proteins [HAPS] and CCAAT box binding factors [CBFs]) are sequence-specific transcription factors with histidine-like subunits that are uniquely characterized by their binding to DNA at the CCAAT site as a heterotrimeric complex consisting of NF-YA, NF- YB and NF-YC, three individual subunits of the protein family [2]. To date, members of the *NF-Y* gene family have been identified in a variety of plants, including *Arabidopsis thaliana *[3], *Oryza sativa *[4], *Prunus persica *[5], and *Populus *[6]. Studies have shown that the *NF-Y* gene family influences flowering in plants [7], improves photosynthetic capacity [8], regulates embryogenesis in *Arabidopsis *[9], and assists in abiotic stress resistance [10–12]. NF-Y family members are involved in plant responses to abiotic stresses, and *ZmNF-YB16* functions by regulating the expression of photosynthesis-related genes to improve the antioxidant capacity of cells and thus achieve drought resistance [13]. In *Lycopersicon esculentum*, *SlNFYA10* negatively regulates the AsA (ascorbic acid) biosynthetic pathway by binding to the CCAAT box in response to oxidative stress [14]. Overexpression of the *GmNFYA5* gene in soybean enhances drought resistance [15]. Although the *NF-Y* gene family has been extensively studied in other species, it has not been reported in ginseng.

Jilin ginseng (*Panax ginseng* C.A. Meyer) is a perennial herb in the *Araliaceae* family that has a cultivation history of at least 4,000 years. The Chinese "Sheng Nong’s Herbal Classic" records in detail that ginseng tastes sweet, can be used as a tonic for the five organs, calms the spirit, fixes the soul, stops panic and palpitations, removes evil spirits, brightens the eyes, makes the mind happy, educates the mind, lightens the body and prolongs life when taken for a long time [16]. Ginseng is not currently used by consumers as a single medicinal plant but as a food and health product [17]. The quantity of wild ginseng can hardly meet the social demand for ginseng products, while the quality of artificially cultivated ginseng is constrained by various factors. Some members of the NF-Y gene family respond to salt stress. We obtained the *NF-Y* gene family members in response to salt stress treatment by screening the adventitious roots of ginseng, which will provide genetic resources for the subsequent improvement of salt stress resistance in ginseng cultivars.

In this study, 40 *PgNF-Y* genes were identified in the Jilin ginseng transcriptome database and classified according to their structural domain information (NF-YA, NF-YB, and NF-YC). Subsequently, we analyzed the evolutionary relationships of the *PgNF-Y* gene family, and conserved motifs and annotated them with GO functions. In addition, expression pattern analysis and coexpression network analysis were performed based on the *PgNF-Y* gene expression data. Finally, the response of PgNF-Y family members to different concentrations of salt was explored. The results of this experiment provide important theoretical information and experimental data on the *NF-Y* gene family for the subsequent study of functional genes in ginseng.

## Materials and methods

### Identification of the *NF-Y* genes in *Panax ginseng*

To maximize data integrity and reliability, we adopted three different approaches to screen members of the *NF-Y* gene family in the Jilin ginseng database containing 248,993 transcripts [18]. First, the hidden Markov model of the NF-Y gene family was downloaded from the PFAM protein family database (http://pfam.xfam.org/), and using PFAM IDs (PF02045, PF00808) as the interrogated sequences, tBlastn was performed in the Jilin ginseng transcriptome. Second, the coding and protein sequences of NF-YA, NF-YB, and NF-YC were downloaded from the Korean Ginseng Genome website (http://ginsengdb.snu.ac.kr/pathway.php); these sequences were used as the interrogation sequences for Blastn and tBlast of the Jilin ginseng transcriptome database at an e-value of 1 × 10^–6^. Finally, 10 protein sequences from the NF-Y family with verified functions were downloaded from GenBank (https://www.ncbi.nlm.nih.gov/) and used as interrogation sequences for tBlastn of the transcriptome of Jilin ginseng. These 10 sequences were from *Arabidopsis thaliana *[10, 19], *Triticum aestivum *[20, 21], *Oryza sativa *[22], *Glycine max *[19], *Solanum tuberosum *[8], *Nicotiana tabacum* [23], and *Zea may*s [11]. Subsequently, the results obtained by the three methods were combined, and after removing duplicate values, the files were used for our preliminary investigation. To exclude some spurious comparison information, we submitted the results to iTAK (http://itak.feilab.net/cgi-bin/itak/index.cgi) for online analysis while maintaining the sequence information regarding NF-Y structural domains. Based on the results of iTAK, all the obtained NF-Y transcripts were named PgNF-Ys. Finally, the obtained transcripts were verified one by one for structural domains by the SMART online tool (http://smart.embl-heidelberg.de/).

### Phylogenetic evolutionary analysis and conserved motifs of the *PgNF-Y* gene family in *Panax ginseng*

We identified 79 transcript sequences with complete open reading frames from 115 *PgNF-Y* gene transcripts based on ORF Finder in NCBI (the remaining 36 transcripts contained only the structural domain information of NF-Y). Based on these results, 12 nucleic acid sequences in the NF-YA, NF-YB, and NF-YC subgroups from *Lycopersicon esculentum*, *Arabidopsis thaliana*, *Oryza sativa*, and *Helianthus annuus* were downloaded from the NCBI database as outgroups for the phylogenetic tree. These nucleic acid sequences were translated into protein sequences. In MEGA-X software [24], the maximum-likelihood (ML) method was chosen to obtain the evolutionary tree of the *PgNF-Y* genes with 1,000 bootstrap replicates to further illustrate the evolutionary relationships between the *PgNF-Y* genes in different species. Finally, the evolutionary tree was optimized by Evolview (https://www.evolgenius.info/evolview/#/treeview). To determine the conserved sequence patterns in the *NF-Y* gene family members in Jilin ginseng, we analyzed the motifs of PgNF-Y transcription factors by the MEME online tool (https://meme-suite.org/meme/doc/cite.html?man_type=web). Finally, the obtained results were visualized by TBtools [25].

### GO (Gene Ontology) annotation and functional categorization of *PgNF-Y* gene transcripts

We used Blast2GO version 6.0.3 [26] to annotate the identified NF-Y transcripts based on Gene Ontology (GO) functional annotation into three major categories: BP (Biological Process), CC (Cellular Component), and MF (Molecular Function). The enrichment of all the nodes where the genes were located was determined by the *Chi-square* test at Level 2. The functions of 109,781 transcripts that were annotated by previous authors were used as reference information [18].

### Expression pattern and network analysis of *PgNF-Y* gene transcripts

Since gene expression is subject to a variety of conditions, we obtained data on the expression of PgNF-Ys in 42 farm cultivars, 14 different tissues of 4-year-old ginseng, and 4-year-old ginseng roots, and we plotted the heatmap in R. Thus, the expression pattern of the *PgNF-Y* genes in Jilin ginseng in time and space was determined. To further investigate the interrelationship between *PgNF-Y* gene expression in 42 farm cultivars, Spearman's correlation coefficients of *PgNF-Y* gene expression were calculated using R, and BioLayout Express ^3D^ version 3.3 software was used to form a visual network of the obtained results.

### Analysis of the response of *PgNF-Y* genes to salt stress

In this experiment, the adventitious roots of Jilin ginseng (*Panax ginseng* C.A. Meyer) were treated with different concentrations of salt in B5 medium (0, 70, 80, 90, and 100 mM NaCl), and the treated adventitious roots were incubated under dark conditions at 22 °C for 30 days.

### Plant materials, RNA isolation, and quantitative real-time PCR analysis

Adventitious root material (0.1 g) treated with different salt concentrations was weighed separately, and total RNA was extracted from ginseng adventitious root tissue using TRIzol (BioTeke, Beijing, China). The HiFiScript gDNA Removal cDNA Synthesis Kit (CWBIO, Beijing, China) was used to reverse transcribe the extracted RNA into cDNA. Actin 1 was selected as the internal reference gene, and according to the instructions for the UltraSYBR One-step RT-qPCR Kit (Low ROX) (CWBIO, Beijing, China), a 7500 Real Time PCR System was used to perform the reaction. The total reaction system was 10 µL, which included UltraSYBR Mixture (Low ROX) at 5 μL, upstream and downstream primers at 0.2 μL each, template at 1 μL, and ddH_2_O at 3.6 μL. qPCR was performed in a thermal cycling system with the following conditions: 95 °C for 10 min and 40 cycles of 95 °C for 15 s, and 60 °C for 60 s. To ensure the accuracy of the results obtained for each treatment, we set up 3 biological replicates and 3 technical replicates. The final results were obtained using the 2^−ΔΔCt^ analysis method.

## Results

### Identification of *PgNF-Y* gene family transcripts

A total of 2,266 transcripts remained after removing duplicates from the NF-Y gene family transcripts obtained by three different methods for interrogating the Jilin ginseng transcriptome database. These transcripts were submitted to iTAK and queried for structural domains by SMART. Finally, 115 transcripts containing the structural domain of the *NF-Y* gene family were obtained. We collected basic information on the *PgNF-Y* gene family members of Jilin ginseng, including transcript ID, gene ID, mRNA sequence information, the number of transcripts with a length ranging from 240–2,624, and the number of amino acids with complete open reading frames (ORFs) ranging from 70–336 (Table S1).

### Motif prediction and phylogenetic analysis of *PgNF-Y* genes

**Fig. 1:**
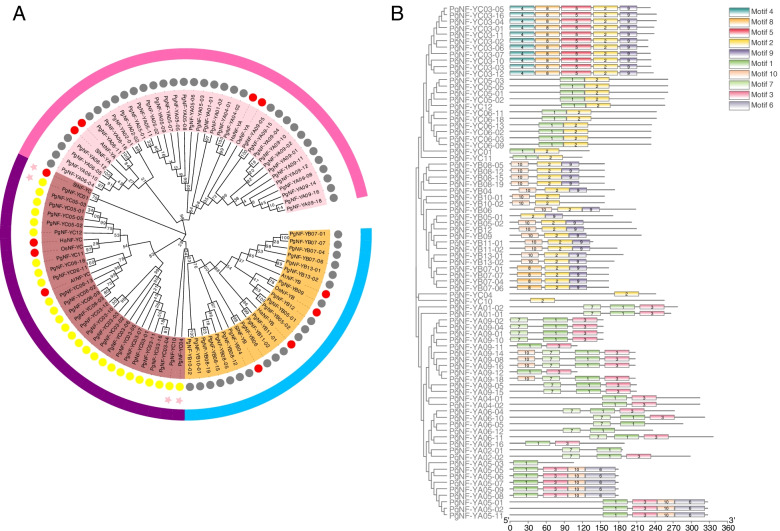
Phylogenetic relationship and conserved motifs of the PgNF-Y proteins. **A** Phylogeny of the *PgNF-Y* gene family. A total of 79 *PgNF-Y* transcripts that had complete ORFs and the longest length among the transcripts alternatively spliced from a gene were used as representatives to construct the phylogeny of the *PgNF-Y* gene family (Table S3). A total of 12 *PgNF-Y* genes from *Arabidopsis thaliana* (At), *Oryza sativa* (Os), *Solanum lycopersicum* (Sl) and *Helianthus annuus* (Ha) were used as outgroup (Table S4). The subfamilies to which the *PgNF-Y* genes belong are indicated by numbers highlighted by red color. The numbers for the branches of the tree are bootstrap confidence out of 1000 replications. **B** Conserved motifs of *PgNF-Y* proteins according to their evolutionary relationship. The conserved motifs of the *PgNF-Y* proteins are indicated by colored boxes. The capital letters indicate the groups of the *PgNF-Y* gene family

Since ginseng originated approximately 100 million years ago in the Cretaceous period, it has a long evolutionary history. We further investigated the evolutionary characteristics of *PgNF-Y* gene family members in Jilin ginseng based on 79 NF-Y gene family members with complete ORFs (the remaining 36 transcripts contained only structural domain information of NF-Y). We explored the evolutionary relationships of the *PgNF-Y* gene family from the perspectives of closely related species, model plants, monocotyledons, and dicotyledons. We selected *Solanum lycopersicum*, *Arabidopsis thaliana*, *Oryza sativa*, and *Helianthus annuus* as outgroups for phylogenetic analysis via the maximum likelihood method (Table S2). Phylogenetic tree analysis showed that the transcripts of the PgNF-YA, PgNF-YB, and PgNF-YC subgroups were all concentrated in specific subclades (except *PgNF-YC04* and *PgNF-YC10*), and the NF-Y members that were distributed in the same subclass had functional similarity. Based on the evolutionary relationship between outgroups and PgNF-Y transcripts, NF-Y members in these species have an evolutionary origin from a common ancestor (Fig. 1A).

To understand the sequence characteristics of the *PgNF-Y* protein, its conserved structural domains were analyzed by the online tool MEME. The results showed that among the 10 motifs analyzed, the number of motifs contained in different subfamily members ranged from 1 to 5 (Fig. 1B). Motif 3, motif 6, and motif 7 were only present in the PgNF-YA subfamily; motif 4, motif 5, and motif 8 were only present in the NF-YC subfamily; and motif 2, motif 9, and motif 10 were commonly found in the NF-YB subfamily. The differences in the number and type of motif in different subfamilies demonstrate that the *NF-Y* gene family is functionally diverse and structurally different.

### GO annotation of *PgNF-Y* gene transcripts

**Fig. 2:**
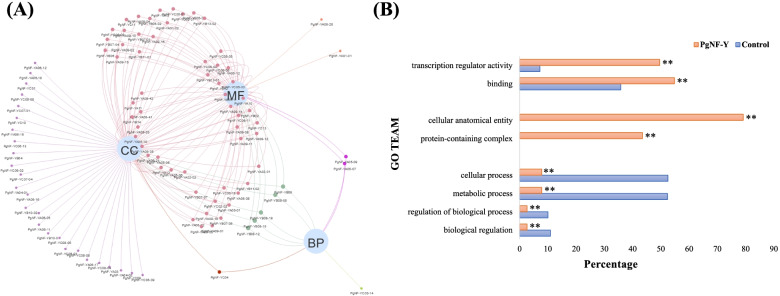
Functional categorization and GO term enrichment of the *PgNF-Y* gene transcripts. **A** Venn network of the *PgNF-Y* gene transcripts among the biological process (BP), molecular function (MF) and cellular component (CC) functional categories. **B** Subcategories (Level 2) into which the *PgNF-Y* transcripts are categorized and GO enrichments. The GO terms of the transcripts expressed in 14 tissues of the four-year-old of ginseng used for identification of the *PgNF-Y* transcripts as the background control for the enrichment analysis. “**” as significant at *P* ≤ *0.01*; “No star” as not significant at *P* ≥ *0.05*

Genes often have more than one function in organisms. To further characterize the properties of *PgNF-Y* genes and gene products, GO annotation of 115 *PgNF-Y* transcripts showed (Fig. 2A, Table S3) that 95 of the 115 transcripts were annotated to any at least one of the three major functions: 9 were annotated to Biological Process, 91 to Cellular Component and 67 to Molecular Function. There were 30 transcripts annotated to only one function, 61 transcripts annotated to two major functions, and 5 transcripts annotated to three major functions (*PgNF-YB08-05*, *PgNF-YB08-12*, *PgNF-YB08-15*, *PgNF-YB08-19*, and *PgNF-YB09*). At Level 2, 4 sublevels (cellular process, metabolic process, regulation of biological process, and biological regulation) were enriched in BP, 2 sublevels (cellular anatomical entity and protein-containing complex) were enriched in CC, and 2 sublevels (transcription regulator activity and binding) were enriched in MF. The results of 109,781 previously annotated transcripts from the Jilin ginseng transcriptome data were used as a reference, and 115 PgNF-Y transcripts were interrogated. The obtained results were subjected to the *chi-square* test in SPSS version 23.0 software, and the results showed that all eight sublevels obtained from interrogated gene enrichment were significantly different from those for reference genes (*P* ≤ *0.05*). The cellular anatomical entity and the protein-containing complex were not annotated in the reference genes (Fig. 2B).

### Expression characteristics of the *PgNF-Y* gene transcripts

**Fig. 3:**
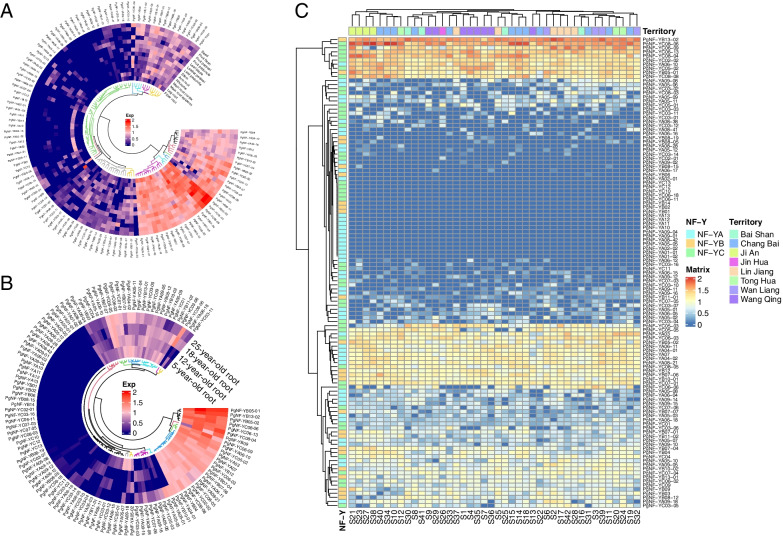
Heatmaps analysis spatiotemporal expression patterns of *PgNF-Y* transcripts in *Panax ginseng*. **A** The *PgNF-Y* genes expressed in the 14 different tissues of 4-year-old ginseng. **B** The *PgNF-Y* genes expressed in the 4 different ages (5, 12, 18, 25 years) of ginseng roots. **C** The *PgNF-Y* genes expressed in the 42 farm cultivars of 4-year-old ginseng roots

To investigate the expression pattern of the *PgNF-Y* gene family from a temporal and spatial perspective, we retrieved *PgNF-Y* gene expression data from 4-year-old Jilin ginseng Damaya roots from the database containing 42 farm cultivars (S1—S42) and 14 different tissues (stem, fiber root, fruit peduncle, main root epiderm, fruit pedicel, rhizome, leaf peduncle, arm root, leaflet pedicel, leg root, leaf blade, fruit flesh, main root cortex, and seed), as well as four ginseng root samples of various ages (5, 12, 18, and 25 years) of *PgNF-Y* gene expression data (Table S4). The results of the heatmap of the roots of 42 farm cultivars showed that 96 *PgNF-Y* transcripts (85%) were expressed in at least one cultivar and 36 transcripts (31%) were expressed in all 42 cultivars (Fig. 3C). *PgNF-YB13-02* and *PgNF-YC08-06* had higher gene expression in 42 farm cultivar, and 18 transcripts were not expressed in any of the cultivar. The heatmap results of 14 tissue from four-year-old ginseng showed that 110 *PgNF-Y* transcripts (96%) were expressed in at least one tissue, and 41 transcripts (36%) were expressed in all 14 tissues (Fig. 3A). *PgNF-YB13-02*, *PgNF-YC08-04*, and *PgNF-YC08-06* were expressed at relatively high levels, and only 5 transcripts were not expressed in any tissue. The heatmap of gene expression in roots of plants at four different ages showed that 85 PgNF-Y transcripts (74%) appeared in at least one age stage and 48 PgNF-Y transcripts (42%) were expressed in all four age stages (Fig. 3B). Among them, *PgNF-YB05-01*, *PgNF-YB13-02*, *PgNF-YB05-02*, *PgNF-YC08-06*, *PgNF-YC06-13*, and *PgNF-YC08-04* were highly expressed, and 30 transcripts were not expressed across the four different ages.

**Fig. 4:**
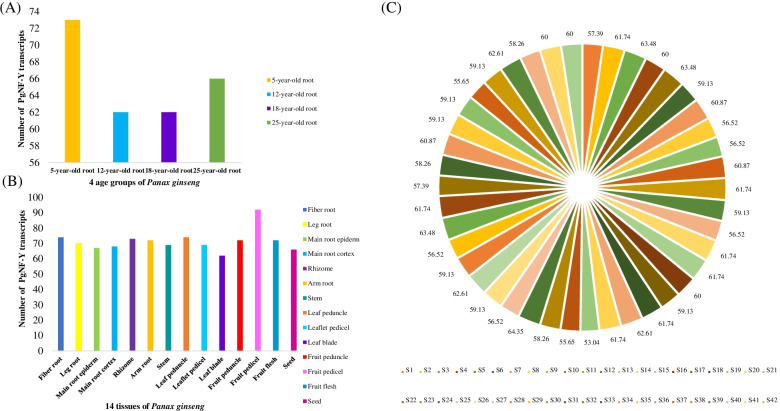
Numbers of the *PgNF-Y* gene transcripts expressing across tissues, the roots of differently aged plants, and genotypes. **A** The histogram of PgNF-Y transcripts expressed in four age groups. **B** The histogram of PgNF-Y transcripts expressed in 14 tissues. **C** Percentage of the *PgNF-Y* gene transcripts expressing in four-year-old roots of different numbers of genotypes

To further understand the expression trends of the *PgNF-Y* genes in Jilin ginseng, the expression of *PgNF-Y* transcripts was analyzed in the roots of 42 farm cultivars, 14 different tissues, and the roots of plants at 4 different ages. In the bar graphs showing the results for the roots of plants at four different ages (Fig. 4A), *PgNF-Y* tended to be more highly expressed in 5-year-old ginseng roots and less expressed in 12-year-old and 18-year-old ginseng roots. In the bar graph of the 14 different tissues within ginseng (Fig. 4B), the PgNF-Y transcripts tended to be expressed mostly in the fruit pedicel, while the leaf blade showed the lowest amount of PgNF-Y expression. Among the 42 farm cultivars of ginseng, the number of PgNF-Y transcripts expressed accounted for 53.04%—63.48% of the total PgNF-Y transcripts.

**Fig. 5:**
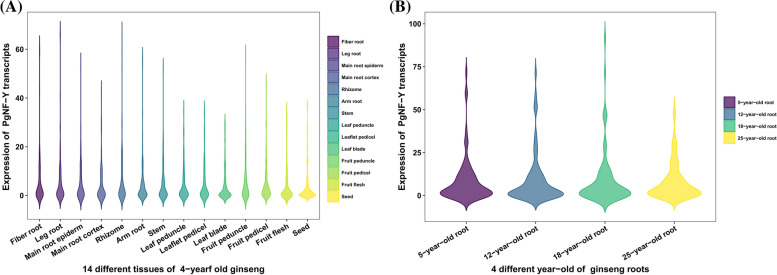
The expression pattern of *PgNF-Y* gene transcripts in *Panax ginseng*. **A** The violinplot of all *PgNF-Y* genes expression in different tissues of ginseng. **B** The violinplot of all *PgNF-Y* genes expression in different ages roots of ginseng

To better understand the general level of *PgNF-Y* gene expression in 14 different tissues and 4 different ages, violin plots of gene expression versus tissue and age were plotted (Fig. 5A and B). The results showed that *PgNF-Y* gene expression was lower in the main root cortex and leaf blade and higher in the fruit peduncle and fruit pedicel; the expression of *PgNF-Y* genes was relatively consistent across the four different ages. Based on this analysis of the expression pattern of *PgNF-Y* transcripts, we found that the expression of *PgNF-Y* genes in Jilin ginseng was influenced by geographical factors, tissues and plant age.

### Coexpression network of* PgNF-Y* gene transcripts

**Fig. 6:**
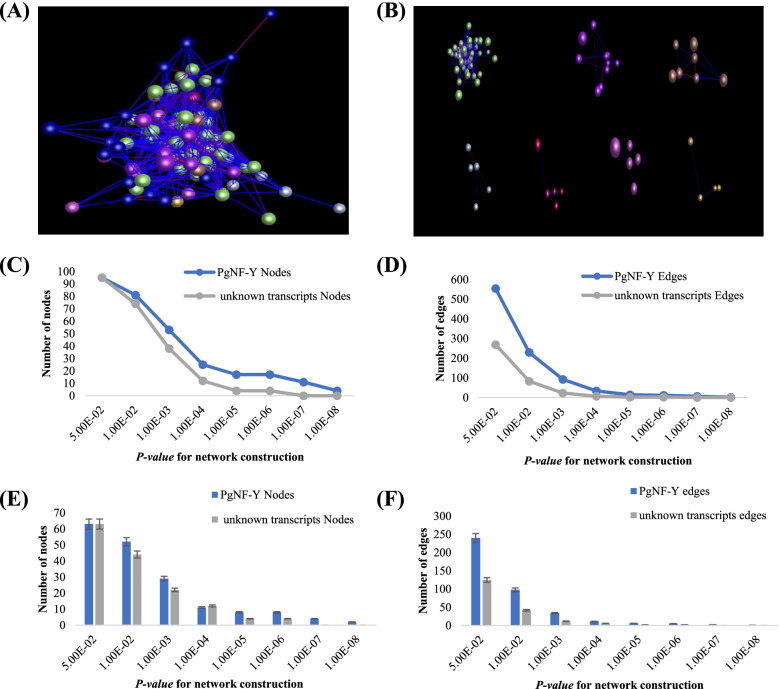
Network analysis of the *PgNF-Y genes* expressed in the 4-year-old roots of 42 farm cultivars. **A** The co-expression network constructed from the 96 *PgNF-Y* transcripts. The network was constructed at *P* ≤ *5.0E-01*. **B** The three clusters constituting the network. Different clusters are indicated by different colors. **C** Tendency that these *PgNF-Y* form a network, with the randomly-selected ginseng unknown genes as controls: variation in number of nodes. **D** Tendency that these *PgNF-Y* transcripts form a network, with the randomly-selected ginseng unknown genes as controls: variation in number of edges. **E** Statistical analysis of variation in number of nodes in the network. **F** Statistical analysis of variation in number of edges in the network. Different capital letters, significant at *P* ≤ *0.01*. Error bar, standard deviation for 20 replications

To investigate whether there is a relationship between *PgNF-Y* transcripts among different genotypes, we performed a coexpression network analysis of *PgNF-Y* transcripts from 42 farm cultivars. We selected 96 of these transcripts for coexpression network analysis (the remaining 19 transcripts were not expressed in any of the 42 farm cultivars). The coexpression network results showed that at *P* ≤ *5.0E-02*, the 96 transcripts formed a coexpression network containing 95 nodes with 554 edges (Fig. 6A and B). The network contains 7 clusters. As the *P value* gradually reduced to *1.0E-08*, the *PgNF-YA09-16* gene remained strongly correlated with *PgNF-YA09-08*, *PgNF-YA09-14*, and *PgNF-YA09-15*. To explore the closeness of the formed networks, we randomly selected 96 transcripts in database A as negative controls and constructed a coexpression network. The results showed (Fig. 6C and D) that the *PgNF-Y* transcripts formed 95 nodes and 269 edges compared to the randomly selected transcripts at *P* ≤ *5.0E-02*, and the number of nodes and edges of the unknown transcripts was 0 when the *P* value gradually decreased to *1.00E-07*. Therefore, we determined that the *PgNF-Y* transcripts form a tighter regulatory network than the negative controls. To further determine the rigor of the regulatory network, we selected two-thirds of the randomly selected *PgNF-Y* transcripts (65) to construct the regulatory network and randomly selected another 65 transcripts as the negative control (Fig. 6E and F). At *P* ≤ *5.0E-02*, the 65 *PgNF-Y* transcripts formed a regulatory network with 63 nodes, and at *P* ≤ *1.0E-08*, the *PgNF-Y* transcripts formed 2 nodes and 1 edge; the number of nodes and edges was 0 for the unknown transcripts at *P* ≤ *1.00E-07*. Thus, the *PgNF-Y* transcripts are more likely to form a coexpression network than the randomly selected transcripts.

### Analysis of the response of *PgNF-Y* genes under salt stress treatment

**Fig. 7:**
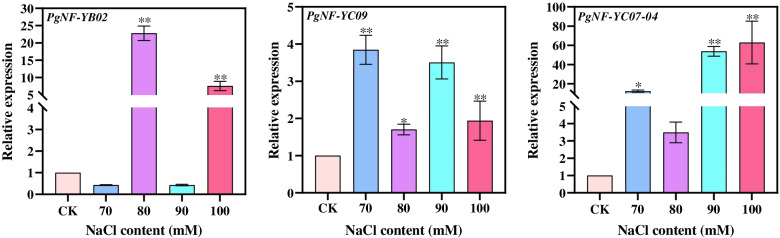
The expressions analysis of *PgNF-YB02*, *PgNF-YC09*, and *PgNF-YC07-04* genes in the roots treated with salt stresses using the qRT-PCR. The 2^−△△^.^Ct^ method was used to evaluate the relative expression, and the expression levels of genes in the control were defined as “1”. The values are presented as the means of three replicates. “*” as significant at *P* ≤ *0.05*, “**” as significant at *P* ≤ *0.01*

Ginseng grown in the wild is subject to a variety of factors, and gene expression in a specific environment is often used to predict gene function. To verify the response of *PgNF-Y* genes to salt stress, we downloaded the known salt tolerance-related NF-Y nucleic acid sequences from the NCBI database and used local BLAST to initially screen three salt resistance-related genes (*PgNF-YB02*, *PgNF-YC09*, and *PgNF-YC07-04*) from 115 *PgNF-Y* gene transcripts. The results of fluorescence quantitative PCR showed that all three *PgNF-Y* genes were upregulated in ginseng adventitious roots under different salt stress treatments (Fig. 7). Under salt stress, the expression of the *PgNF-YC07-04* gene was significantly increased in ginseng adventitious roots compared with the control. This result further indicates that the *PgNF-YC07-04* gene has a strong response to salt stress and plays an important role in the biological process of salt stress resistance in ginseng. This result also provides genetic resources for the study of salt stress resistance and gene breeding in Jilin ginseng.

## Discussion

The NF-Y gene family is prevalent in plants. Therefore, the NF-Y transcription factor family has been extensively studied in plants, including *Arabidopsis thaliana *[27], *Glycine max *[7], *Triticum aestivum *[28], and *Lotus japonicus *[29]. The functional diversity of *NF-Y* gene families has been well demonstrated by targeting specific genes in the *NF-Y* gene family. In this study, 40 *NF-Y* gene family members were identified in Jilin ginseng, compared to 36 *NF-Y* members in *Arabidopsis*, 19 NF-Y members in *Citrullus lanatus*, 24 NF-Y members in *Prunus persica*, and 52 NF-Y members in *Populus simonii*. We found that the number of *NF-Y* gene family members in plants does not seem to differ between herbaceous and woody plants. Polyploidy or whole genome duplication (WGD) is a common phenomenon in angiosperms [30], and the NF-Y transcription factor family in ginseng may have expanded as a result.

Genome sequencing has shown that most of the genes specifying core biological functions are common to all eukaryotes [26]. Multiple potential functions exist for PgNF-Y transcripts. Previous studies have shown that NF-Y transcription factors are involved in the regulation of biological processes such as flowering [31], photomorphogenesis [32], and abiotic stress [10] in *Arabidopsis* by binding to the CCAAT box [33]. According to the GO annotation results, 67 *PgNF-Y* transcripts were annotated to molecular functions (binding and transcription regulator activity); therefore, these genes are likely to bind the CCAAT box of other genes to regulate the growth and development of Jilin ginseng. Nine *PgNF-Y* transcripts were annotated to biological processes, and 91 *PgNF-Y* transcripts were annotated to cellular components, suggesting that *PgNF-Y* genes act not only as a functional gene but also as structural genes in Jilin ginseng. In summary, the *PgNF-Y* transcripts are functionally diverse in Jilin ginseng.

Clarifying the expression pattern of *PgNF-Y* transcripts is helpful for exploring the function of these transcripts. From the analysis of the expression levels of *PgNF-Y* transcripts in the 42 farm cultivars, the relatively close expression patterns of 115 *PgNF-Y* transcripts suggest that they are widely expressed in Jilin ginseng. The *PgNF-YB13-02* and *PgNF-YC08-06* genes had high expression levels in the 42 farm cultivars; therefore, these two genes may be housekeeping genes in Jilin ginseng and thus maintain the basic life activities of the plant. The expression of *PgNF-Y* genes in 14 different tissues of 4-year-old ginseng showed that their expression was tissue specific, and *PgNF-YA12*, *PgNF-YA13*, *PgNF-YB01*, *PgNF-YB08-15*, *PgNF-YC10*, and *PgNF-YC12* were expressed only in the fruit pedicel, suggesting that these five PgNF-Y transcripts may be involved in the development of ginseng fruit pedicels. The *PgNF-YC13* gene was expressed only in leg roots and may be related to their development in ginseng. The median expression levels of the *PgNF-Y* gene in the leaf blade and fruit pedicel were higher than that in other tissues, indicating that *PgNF-Ys* are likely to be involved in ginseng fruit and flower development. In ginseng roots of plants at different ages, approximately 42% of the *PgNF-Y* transcripts were expressed at different times, and approximately 13% of the *PgNF-Y* transcripts were expressed only in a specific year. The median expression levels of *PgNF-Y* genes across the four ages was fairly consistent, indicating that the expression levels of the *PgNF-Y* gene family members did not increase with growth year. The specific expression of *PgNF-Y* transcripts at four different ages suggested that not all *PgNF-Y* transcripts were constitutively expressed in Jilin ginseng, and some transcripts might be induced to be expressed in response to changes in external conditions such as soil and climate.

The NF-Y transcription factor is a heterotrimer formed by evolutionarily conserved subunits: NF-YA, NF-YB, and NF-YC [34]. NF-YB and NF-YC contain a histone folding domain (HFD), and NF-YA binds to the NF-YB/NF-YC dimer [35]. There are 30 predicted NF-Y members in the Arabidopsis genome; in theory, this could result in approximately 1,000 heterotrimeric combinations [36]. In the coexpression network of 42 farm cultivars, we found that PgNF-Y formed some small clusters with close relationships at *P* ≤ *5.0E-02*, suggesting that such heterologous trimers may also be formed in Jilin ginseng. However, this result needs to be verified using yeast two-hybrid and yeast three-hybrid techniques.

The cultivation time for both garden and forest ginseng is usually 5–7 years. During the long cultivation cycle, the soil environment is one of the key factors controlling the quality of ginseng [37]. A recently identified salt tolerance gene, *NF-YC13,* was found in indica rice [38]; the *TaNF-YA10-1* gene was isolated from the salt-tolerant wheat variety SR3; and overexpression of the *NF-YA10* gene in *Arabidopsis thaliana* regulates the response of *Arabidopsis* to salt stress [21]. In this paper, our fluorescence quantitative PCR results showed that all three transcripts, *PgNF-YB02*, *PgNF-YC09*, and *PgNF-YC07-04,* in ginseng responded to salt stress. This result also indicates that the *NF-Y* gene family is prevalent in plants responding to salt stress.

## Conclusion

In this study, we screened 40 *PgNF-Y* genes from Jilin ginseng and analyzed their evolution, structure, function, expression pattern and coexpression network to verify the response of *PgNF-Y* genes to salt stress treatment. The results of the above analysis showed that *PgNF-Y* gene family members are functionally diverse and exhibit tissue specificity, time specificity, and interspecies differences in their expression patterns. *PgNF-Y* gene family members act synergistically with each other in the plant. Moreover, the *PgNF-Y* gene family plays an important role in the plant response to salt stress. This study further demonstrates the role of the *NF-Y* gene family in the response to salt stress and provides a theoretical basis for the genetic breeding of ginseng.

## Supplementary Information

**Additional file 1: Table S1.** Basic information of the PgNF-Y gene family

**Additional file 2: Table S2.** The identified genes used as evolutionary controls for the PgNF-Y gene phylogenetic analysis.

**Additional file 3: Table S3.** The classification, annotation and GO functional categorization of the PgNF-Y gene transcripts

**Additional file 4: Table S4.** The expressions of the PgNF-Y gene transcripts in 14 tissues, 42 farm cultivar roots and 4 ages roots (TPM).

## Data Availability

All data analyzed during this study are included in the supplementary information files, and genotypic data have been deposited in the Sequence Read Archive (https://www.ncbi.nlm.nih.gov/sra) to NCBI under BioProject RJNA302556.

## References

[1] Myers ZA, Holt BF (2018). NUCLEAR FACTOR-Y: still complex after all these years?. Curr Opin Plant Biol.

[2] Petroni K, Kumimoto RW, Gnesutta N, Calvenzani V, Fornari M, Tonelli C, Holt BF, Mantovani R (2012). The promiscuous life of plant NUCLEAR FACTOR Y transcription factors. Plant Cell.

[3] Feng C, Wang Y, Sun Y, Peng X, Zhang X, Zhou X, Jiao J, Zhai Z, Xiao Y, Wang W (2021). Expression of the malus sieversii NF-YB21 encoded gene confers tolerance to osmotic stresses in arabidopsis thaliana. Int J Mol Sci.

[4] Yang W, Lu Z, Xiong Y, Yao J (2017). Genome-wide identification and co-expression network analysis of the OsNF-Y gene family in rice. The Crop Journal.

[5] Li M, Li G, Liu W, et al. Genome-wide analysis of the NF-Y gene family in peach (Prunus persica L.). BMC Genomics. 2019;20:612. 10.1186/s12864-019-5968-7. 10.1186/s12864-019-5968-7 PMC6660701 31349783

[6] Liu R, Wu M, Liu HL, Gao YM, Chen J, Yan HW, Xiang Y (2021). Genome-wide identification and expression analysis of the NF-Y transcription factor family in Populus. Physiol Plant.

[7] Mallano AI, Li W, Tabys D, Chao C, Yang Y, Anwar S, Almas HI, Nisa ZU, Li Y (2021). The soybean GmNFY-B1 transcription factor positively regulates flowering in transgenic Arabidopsis. Mol Biol Rep.

[8] Xuanyuan G, Lu C, Zhang R, Jiang J (2017). Overexpression of StNF-YB3.1 reduces photosynthetic capacity and tuber production, and promotes ABA-mediated stomatal closure in potato (Solanum tuberosum L.). Plant Sci.

[9] Gaj MD, Zhang S, Harada JJ, Lemaux PG (2005). Leafy cotyledon genes are essential for induction of somatic embryogenesis of Arabidopsis. Planta.

[10] Zhang T, Zhang D, Liu Y, Luo C, Zhou Y, Zhang L (2015). Overexpression of a NF-YB3 transcription factor from Picea wilsonii confers tolerance to salinity and drought stress in transformed Arabidopsis thaliana. Plant Physiol Biochem.

[11] Su H, Cao Y, Ku L, Yao W, Cao Y, Ren Z, Dou D, Wang H, Ren Z, Liu H (2018). Dual functions of ZmNF-YA3 in photoperiod-dependent flowering and abiotic stress responses in maize. J Exp Bot.

[12] Li WX, Oono Y, Zhu J, He XJ, Wu JM, Iida K, Lu XY, Cui X, Jin H, Zhu JK (2008). The Arabidopsis NFYA5 transcription factor is regulated transcriptionally and posttranscriptionally to promote drought resistance. Plant Cell.

[13] Wang B, Li Z, Ran Q, Li P, Peng Z, Zhang J (2018). ZmNF-YB16 overexpression improves drought resistance and yield by enhancing photosynthesis and the antioxidant capacity of maize plants. Front Plant Sci.

[14] Chen W, Hu T, Ye J, Wang B, Liu G, Wang Y, Yuan L, Li J, Li F, Ye Z (2020). A CCAAT-binding factor, SlNFYA10, negatively regulates ascorbate accumulation by modulating the D-mannose/L-galactose pathway in tomato. Hortic Res.

[15] Bello BK, Hou Y, Zhao J, Jiao G, Wu Y, Li Z, Wang Y, Tong X, Wang W, Yuan W (2019). NF-YB1-YC12-bHLH144 complex directly activates Wx to regulate grain quality in rice (Oryza sativa L). Plant Biotechnol J..

[16] Kuang H. Chiemistry of Chinese materia medica. China: China Traditional Chinese Medicine Publishing House; 2003–1. (in Chinese)

[17] Baeg IH, So SH (2013). The world ginseng market and the ginseng (Korea). J Ginseng Res.

[18] Wang K, Jiang S, Sun C, Lin Y, Yin R, Wang Y, Zhang M (2015). The spatial and temporal transcriptomic landscapes of Ginseng, Panax ginseng C. A Meyer Sci Rep.

[19] Ma XJ, Yu TF, Li XH, Cao XY, Ma J, Chen J, Zhou YB, Chen M, Ma YZ, Zhang JH (2020). Overexpression of GmNFYA5 confers drought tolerance to transgenic Arabidopsis and soybean plants. BMC Plant Biol.

[20] Yadav D, Shavrukov Y, Bazanova N, Chirkova L, Borisjuk N, Kovalchuk N, Ismagul A, Parent B, Langridge P, Hrmova M (2015). Constitutive overexpression of the TaNF-YB4 gene in transgenic wheat significantly improves grain yield. J Exp Bot.

[21] Ma X, Zhu X, Li C, Song Y, Zhang W, Xia G, Wang M (2015). Overexpression of wheat NF-YA10 gene regulates the salinity stress response in Arabidopsis thaliana. Plant Physiol Biochem.

[22] Kim SK, Park HY, Jang YH, Lee KC, Chung YS, Lee JH, Kim JK (2016). OsNF-YC2 and OsNF-YC4 proteins inhibit flowering under long-day conditions in rice. Planta.

[23] Sun X, Lian H, Liu X, Zhou S, Liu S (2017). The garlic NF-YC gene, AsNF-YC8, positively regulates non-ionic hyperosmotic stress tolerance in tobacco. Protoplasma.

[24] Kumar S, Stecher G, Li M, Knyaz C, Tamura K (2018). MEGA X: molecular evolutionary genetics analysis across computing platforms. Mol Biol Evol.

[25] Chen C, Chen H, Zhang Y, Thomas HR, Frank MH, He Y, Xia R (2020). TBtools: an integrative toolkit developed for interactive analyses of big biological data. Mol Plant.

[26] Conesa A, Gotz S, Garcia-Gomez JM, Terol J, Talon M, Robles M (2005). Blast2GO: a universal tool for annotation, visualization and analysis in functional genomics research. Bioinformatics.

[27] Gusmaroli G, Tonelli C, Mantovani R (2001). Regulation of the CCAAT-Binding NF-Y subunits in Arabidopsis thaliana. Gene.

[28] Ma X, Li C, Wang M (2015). Wheat NF-YA10 functions independently in salinity and drought stress. Bioengineered.

[29] Soyano T, Kouchi H, Hirota A, Hayashi M (2013). Nodule inception directly targets NF-Y subunit genes to regulate essential processes of root nodule development in Lotus japonicus. PLoS Genet.

[30] Van PY, Mizrachi E, Marchal K (2017). The evolutionary significance of polyploidy. Nat Rev Genet.

[31] Kumimoto RW, Adam L, Hymus GJ, Repetti PP, Reuber TL, Marion CM, Hempel FD, Ratcliffe OJ (2008). The Nuclear Factor Y subunits NF-YB2 and NF-YB3 play additive roles in the promotion of flowering by inductive long-day photoperiods in Arabidopsis. Planta.

[32] Myers ZA, Kumimoto RW, Siriwardana CL, Gayler KK, Risinger JR, Pezzetta D, Holt Iii BF (2016). NUCLEAR FACTOR Y, Subunit C (NF-YC) transcription factors are positive regulators of photomorphogenesis in Arabidopsis thaliana. PLoS Genet.

[33] Gusmarolia G, Tonelli C, Mantovani R (2001). Regulation of the CCAAT-Binding NF-Y subunits in Arabidopsis thaliana. Gene.

[34] Gnesutta N, Saad D, Chaves-Sanjuan A, Mantovani R, Nardini M (2017). Crystal structure of the Arabidopsis thaliana L1L/NF-YC3 histone-fold dimer reveals specificities of the LEC1 family of NF-Y subunits in plants. Mol Plant.

[35] Nardone V, Chaves-Sanjuan A, Nardini M (2017). Structural determinants for NF-Y/DNA interaction at the CCAAT box. Biochim Biophys Acta Gene Regul Mech.

[36] Zhao H, Wu D, Kong F, Lin K, Zhang H, Li G (2016). The Arabidopsis thaliana Nuclear Factor Y transcription factors. Front Plant Sci.

[37] Paek KY, Murthy HN, Hahn EJ, Zhong JJ (2009). Large scale culture of ginseng adventitious roots for production of ginsenosides. Adv Biochem Eng Biotechnol.

[38] Manimaran P, Venkata Reddy S, Moin M, Raghurami Reddy M, Yugandhar P, Mohanraj SS, Balachandran SM, Kirti PB (2017). Activation-tagging in indica rice identifies a novel transcription factor subunit, NF-YC13 associated with salt tolerance. Sci Rep.

